# OMRT-9. Effect of Pre-Operative Stereotactic Radiosurgery on Brain Metastasis: Analysis of DNA and RNA Genomic Profiles from Phase-II Clinical Trial NCT03398694

**DOI:** 10.1093/noajnl/vdab070.034

**Published:** 2021-07-05

**Authors:** Jack Shireman, Wei Huff, Gina Monaco, Namita Agrawal, Gordon Watson, Mahua Dey

**Affiliations:** 1 University of Wisconsin, Madison, WI, USA; 2 University of Indiana, Indianapolis, IN, USA

## Abstract

**Background:**

With improved systemic therapy that has limited impact on the intracranial compartment, the incidence of brain metastasis (BM) from solid cancers is rising and negatively impacting patient’s overall survival (OS). Treatment varies based on presentation, however, for patients with <4 symptomatic BMs current clinical practice involves surgical resection followed by stereotactic radiosurgery (SRS) to the resection cavity. Post-operative SRS is associated with increased risk of leptomeningeal disease (LMD) and local recurrence in the follow-up period. We hypothesize that pre-operative SRS will decrease the incidence of LMD as well as local recurrence and increase patient’s OS by delivering a lethal dose of radiation to tumor cells before they are disturbed by surgical resection. In a Phase II clinical trial (NCT03398694) we are treating patients with 1–4 symptomatic BMs with pre-operative SRS while collecting DNA and RNA sequencing data from core and peripheral edges of the resected tumor to examine the genomic effects of SRS on tumor.

**Methods:**

Post-SRS resected tumor specimens were divided into two groups: ‘center’ and ‘periphery’ with respect to the center of SRS treatment with periphery within 50% isodose line. Previously resected untreated BMs were used as control. DNA and RNA were isolated from all samples for sequencing.

**Conclusions:**

Our initial analyses show that pre-treatment with SRS, results in significant genomic changes at DNA and RNA levels throughout the tumor, in both center as well as periphery. Furthermore, significant transcriptomic differences were noted among matched samples between the central and peripheral SRS locations implicating differential effect of SRS dosing within a tumor. Initial gene ontological analysis on non-small cell lung cancer samples demonstrated an overexpression of WNT and BMP signaling pathways (p <.001, p<.01). These pathways are typically involved in neuronal development, hinting that adaptation to the brain microenvironment was occurring post SRS treatment.

